# High prevalence of lower limb atherosclerosis is linked with the gut–liver axis in patients with primary biliary cholangitis

**DOI:** 10.1111/liv.15463

**Published:** 2022-11-11

**Authors:** Francesca Romana Ponziani, Antonio Nesci, Camilla Caputo, Lucia Salvatore, Anna Picca, Federica Del Chierico, Francesco Paroni Sterbini, Emanuele Marzetti, Angela Di Giorgio, Luca Santoro, Lorenza Putignani, Antonio Gasbarrini, Angelo Santoliquido, Maurizio Pompili

**Affiliations:** ^1^ Internal Medicine and Gastroenterology–Hepatology Unit Fondazione Policlinico Universitario Agostino Gemelli IRCCS Rome Italy; ^2^ Department of Cardiovascular Sciences, Angiology and Noninvasive Vascular Diagnostics Unit Fondazione Policlinico Universitario Agostino Gemelli IRCCS Rome Italy; ^3^ Department of Geriatrics, Neuroscience and Orthopedics Fondazione Policlinico Universitario Agostino Gemelli IRCCS Rome Italy; ^4^ Multimodal Laboratory Medicine Research Area Unit of Human Microbiome, Bambino Gesù Children's Hospital IRCCS Rome Italy; ^5^ Department of Medicine and Surgery LUM University Casamassima Italy; ^6^ Phase 1 Unit Fondazione Policlinico Universitario Agostino Gemelli IRCCS Rome Italy; ^7^ Department of Diagnostic and Laboratory Medicine, Unit of Microbiology and Diagnostic Immunology, Unit of Microbiomics and Multimodal Laboratory Medicine Research Area, Unit of Human Microbiome Bambino Gesù Children's Hospital, IRCCS Rome Italy; ^8^ Catholic University Rome Italy

**Keywords:** atherosclerosis, gut microbiota, inflammation, NAFLD, PBC

## Abstract

**Background and Aims:**

Hypercholesterolemia is frequent in people with primary biliary cholangitis (PBC); however, it does not seem to confer an increased risk of cardiovascular disease. We aimed to evaluate the prevalence of peripheral arterial disease in PBC women and its association with the gut–liver axis and systemic inflammation.

**Methods:**

Thirty patients affected by PBC and hypercholesterolemia were enrolled, with equal‐sized groups of women with non‐alcoholic fatty liver disease (NAFLD) and healthy controls (CTRL). All patients underwent Doppler ultrasound examination of peripheral arteries, assessment of flow‐mediated dilation, quantification of circulating cytokines and vasoactive mediators and characterization of the gut microbiota.

**Results:**

PBC patients had a higher prevalence of lower extremity arterial disease (LEAD) defined as atherosclerotic plaques in any of femoral, popliteal and/or tibial arteries compared with both NAFLD and CTRL women (83.3% vs. 53.3% and 50%, respectively; *p* = .01). Factors associated with LEAD at univariate analysis were VCAM‐1 (*p* = .002), ICAM‐1 (*p* = .003), and TNF‐alpha (*p* = .04) serum levels, but only VCAM‐1 (OR 1.1, 95% CI 1.0–1.1; *p* = .04) and TNF‐alpha (OR 1.12, 95% CI 0.99–1.26; *p* = .04) were confirmed as independent predictors in the multivariate model. Gut microbiota analysis revealed that *Acidaminococcus* (FDR = 0.0008), *Bifidobacterium* (FDR = 0.001) and *Oscillospira* (FDR = 0.03) were differentially expressed among groups. *Acidaminococcus*, which was increased in PBC, was positively correlated with TNF‐alpha serum levels. Down‐regulation of metabolic pathways linked to fatty acid and butyrate metabolism, glyoxylate metabolism and branched‐chain amino acids degradation was found in the functional gut metagenome of PBC women.

**Conclusions:**

LEAD is common in patients affected by PBC and is associated with inflammatory markers and alterations in the gut–liver axis.

**LAY SUMMARY**

• Hypercholesterolemia is common in people affected by primary biliary cholangitis (PBC) but seems not to be associated with cardiovascular events or carotid atherosclerosis.

• PBC patients have a higher prevalence of lower extremity arterial disease (LEAD), which is associated with high‐serum vasoactive and inflammatory mediators.

• The gut microbiota of PBC women is enriched in *Acidaminococcus*, which is correlated with serum inflammatory mediators and express metabolic pathways in fatty acid metabolism and inflammatory/pro‐oxidative responses that can be involved in vascular damage.

## 1 INTRODUCTION

Hypercholesterolemia is a key factor in the pathogenesis of atherosclerosis and cardiovascular risk stratification, and is associated with cholestatic diseases such as primary biliary cholangitis (PBC).^1^ The mechanisms leading to increased blood cholesterol in PBC patients are complex and include greater hepatic production consequent to impaired intestinal absorption and defective biliary cholesterol excretion, decreased hepatic uptake of low‐density lipoproteins (LDLs) and impaired activity of lecithin cholesterol acyltransferase.^2^

Although hypercholesterolemia is frequent in people affected by PBC, data on cardiovascular risk are contrasting. Although recent large‐cohort studies^3^ reported an increased incidence of cardiovascular events and atherosclerosis in PBC patients, others showed opposite results, in particular regarding the carotid district.^4^, ^5^ Various protective factors have been called into question, including increased serum levels of lipoprotein‐X, adiponectin and high‐density lipoproteins (HDLs) and reduction of Lp(a) serum levels and visceral fat.^2^ A local and systemic proinflammatory milieu is critical for the development of atherosclerosis, and, recently, the gut microbiota has been recognized to exert a pivotal role in this scenario through metabolic and inflammatory pathways.^6^ However, little is known about the connection between these factors and alterations of the peripheral vascular system in PBC.

This study aimed to evaluate the prevalence of peripheral atherosclerosis in PBC women, compared with patients with non‐alcoholic fatty liver disease (NAFLD), who frequently show hypercholesterolemia and have an increased prevalence of cardiovascular disease, and healthy controls (CTRL). In addition, our analysis aimed at identifying possible correlations between inflammation, gut–liver axis and vascular disease.

## 2 PATIENTS AND METHODS

In this prospective study, we included 90 women, 30 affected by PBC, 30 affected by NAFLD and 30 CTRL, matched by age, LDL cholesterol and with a low cardiovascular risk according to the Framingham risk score.^7^ PBC or NAFLD women were referred to the liver disease outpatient clinic of the Policlinico Universitario Agostino Gemelli IRCCS (Rome, Italy), whereas healthy controls were chosen among volunteers and medical personnel. PBC was diagnosed at least 5 years before the enrollment in this study in patients with elevated serum levels of alkaline phosphatase (ALP) and positive anti‐mitochondria antibodies (AMAs) or specific antinuclear antibodies. In cases with negative antibodies, liver biopsy was performed, and the diagnosis was histologically established.^8^ Only PBC patients treated for at least 12 months with dose‐appropriate (13–15 mg/kg/day) ursodeoxycholic acid (UDCA) and with an adequate response to UDCA according to Paris II criteria^7^ were included. NAFLD was diagnosed by abdominal ultrasound (US) examination.^9^

The following exclusion criteria were adopted: age lower than 40; other concomitant chronic liver diseases (e.g. viral hepatitis, NAFLD, genetic disorders or autoimmune hepatitis); active alcohol intake; previous liver transplantation; diagnosis of advanced fibrosis or liver cirrhosis according to imaging/laboratory/clinical criteria; history of cancer; active hypolipidemic treatment (including nutraceuticals); active alcohol use; active smoking; pregnancy and ongoing treatment with obeticholic acid. To reduce the risk of bias in the analysis of the gut microbiome, only Caucasian women on an omnivorous, Mediterranean, normocaloric diet, and who had not taken antibiotics, probiotics, prebiotics or proton pump inhibitors in the past 6 months were included. We also excluded women with diseases that may affect the composition of gut microbiota (e.g. celiac disease, inflammatory bowel diseases).

At the time of enrollment, demographic data, medical history, anthropometric measures (weight, height) and results of laboratory examinations (platelet count [PLT], total cholesterol, HDL, LDL, triglycerides, aspartate transaminase [AST], alanine transaminase [ALT], ALP, and gamma‐glutamyl transferase [GGT]) were recorded.

For each participant, a serum sample was collected for the analysis of cytokines and vascular mediators, and a stool sample was used for the gut microbiome analysis. To assess the presence of atherosclerotic lesions, participants underwent Doppler ultrasound (DUS) examination of carotid arteries, abdominal aorta and arterial vessels of the lower limbs. A morpho‐functional study of the brachial artery for the evaluation of flow‐mediated dilation (FMD), as expression of the endothelial function, was also performed.

The study was approved by the institutional review board of the Fondazione Policlinico Universitario Agostino Gemelli IRCCS (ID 1769) and was conducted according to the criteria set by the Declaration of Helsinki; all participants provided written informed consent prior to inclusion.

### 2.1 Arterial Doppler ultrasound examination

DUS examination of peripheral arteries and aorta was performed using the Philips EPIQ Elite high‐resolution US scanner (Philips Ultrasound Inc.). A 12–3 MHz linear probe was used to identify atherosclerotic plaques in the carotid district and the lower‐limb arteries (including common, superficial and deep femoral arteries, popliteal and tibial arteries). As for the carotid district, the presence of intima media thickness was defined as the distance from the leading edge of the lumen–intima interface to the leading edge of the media–adventitia interface ≥0.9 mm and ≤1.3 mm, while atherosclerotic lesions were defined as parietal appositions ≥1.4 mm. The abdominal aorta was explored from the diaphragm to the iliac bifurcation, then to the common iliac arteries to the bifurcation and the proximal portion of the internal and external iliac arteries, using a 5–1 MHz convex probe.

Non‐invasive assessment of endothelial function was performed by testing brachial artery reactivity. Briefly, the diameter of the right brachial artery was measured after a supine rest period of 15–20 minutes on a longitudinal section of 5–7 cm above the antecubital fossa. Next, a blood pressure cuff was inflated to a pressure of 250 mmHg for 5 min around the forearm distal to the target area, and then deflated abruptly; at this point, a second scan was performed for 90 s to measure changes in diameter after reactive hyperaemia. FMD, representing endothelium‐dependent dilation, was expressed as a percentage increase over baseline diameter. All examinations were performed by a single experienced sonographer (A.N.) blinded to the clinical characteristics of the subjects, at the same time in the morning (8 a.m.), in a temperature‐controlled room (22°C), after avoiding consumption of vasoactive substances for at least 12 hours (i.e. drugs, caffeine, smoking) and after fasting for at least 8 h.

### 2.2 Analysis of cytokines and vascular mediators

Serum samples were used for the quantification of pro‐inflammatory cytokines and vascular mediators. Levels of interleukin (IL)‐1beta, IL‐6, interferon (IFN)‐gamma and tumour necrosis factor (TNF)‐alpha were determined as part of a panel of 27 inflammatory markers, growth factors and chemokines measured simultaneously through a magnetic bead‐based immunoassay on a Bio‐Plex® System with Luminex xMAP® Technology (#M500KCAF0Y, Bio‐Rad Laboratories Inc.) as previously described.^11^, ^12^ Serum levels of adipsin, adiponectin and adhesion molecules [intercellular adhesion molecule‐1 (ICAM‐1) and vascular adhesion molecule‐1 (VCAM‐1)] were quantified on a Bio‐Plex® System according to the manufacturer's instructions (#171A7002M, Bio‐Rad). A commercially available ELISA kit was used to measure the levels of fibroblast growth factor 19 (FGF19) (DF1900, R&D system).

### 2.3 Collection and analysis of stool samples

Fresh stool samples obtained from a bowel movement of the day of enrollment were immediately frozen at −80°C until analysis.

DNA extraction was performed by QIAmp Fast DNA Stool mini kit (Qiagen, Germany), according to the manufacturer's instructions.

The V3‐V4 variable region of the 16 S rRNA gene (~460 bp) was amplified by using the primer pairs 16 S_F 5 ‘‐ (TCG TCG GCA GCG TCA GAT GTG TAT AAG AGA CAG CCG ACG GGN GGC WGC AG) ‐ 3’ and 16 S_R 5′ ‐ (GTC TCG TGG GCT CGG AGA TGT GTA TAA GAG ACA GGA CTA CHV GGG TAT CTA ATC C) ‐3′ (MiSeq rRNA Amplicon Sequencing protocol, Illumina, San Diego, CA). The PCR reaction was set up using a 2x KAPA HotStart HMI ready mix (KAPA Biosystems Inc.). DNA amplicons were cleaned by AMPure XP beads (Beckman Coulter Inc.). The indexing was obtained by a second amplification step using Nextera grafted and back‐labelled adaptor primers. The final library was cleaned using 50 μl of AMPure XP beads, quantified using the Quant‐iT™ PicoGreen® dsDNA Assay Kit (Thermo Fisher Scientific) and diluted to equimolar concentration (4 nM). Samples were pooled together prior to sequencing on an Illumina MiSeqTM platform according to manufacturer's instructions. Bioinformatic analysis of bacterial 16 S rRNA amplicon data was performed using a combination of the QIIME 1.9.1 software pipeline and the VSEARCH v1.1 pipeline. Paired raw sequences were merged using fastqjoin (perc_max_diff: 15, min_overlpp: 50) and the Q20 library (Qiime). After dereplication and chimera checking, reads were grouped into operational taxonomic units (OTUs) at 97% identity (Vsearch). The taxonomy of each 16 S rRNA gene sequence was assigned using UCLUST against the Greengenes 13.8 database with 97% sequence similarity.

### 2.4 Statistical analysis

Data for continuous variables were expressed as median and interquartile range, while those for categorical variables were recorded as frequency and percentage. Owing to the non‐normal distribution of data, we used Fisher, Wilcoxon and Kruskal–Wallis tests with Bonferroni post‐hoc analysis to assess differences among participant groups. Demographic and clinical data as well as cytokines and adhesion molecules that were significantly associated with lower extremity arterial disease (LEAD) at univariate analysis were selected to construct a multivariate model to identify predictors of atherosclerosis.

For the analysis of the gut microbiota, we evaluated compositional differences among PBC, NAFLD and CTRL women, their correlation with parameters mainly associated with LEAD, and the predicted functional profile.

Specifically, raw data were used to assess differences in alpha diversity between groups. Data were subsequently processed by removing extremely rare taxa (those not seen more than three times in at least 20% of the samples), and normalized by regularized logarithm transformation (rlog). Non‐metric multidimensional scaling (NMDS) on Bray–Curtis distance was used to plot beta diversity and groups were compared by permutational multivariate analysis of variance (PERMANOVA).

Differences in relative abundance were explored using the DESeq2 package, which applies a negative binomial distribution on raw counts normalized by ‘size factors’, taking into account sequencing depth between samples. Comparisons with a log2 fold change (log2FC) higher or lower than ±1.5 and a *q*‐value <.05, after adjusting for multiple comparisons with the Benjamini–Hochberg method to control the false discovery rate (FDR), were considered as statistically significant.

The correlation between LEAD‐associated factors and gut microbiota genera or, if unclassified, families, was assessed by Spearman's coefficient, adjusting p‐values for multiple comparisons as described above.

Finally, functional features of the gut microbiome of PBC women were predicted using Phylogenetic Investigation of Communities by Reconstruction of Unobserved States (PICRUSt), and compared with NAFLD and CTRL groups, correcting p‐values to FDR.

Analyses were performed using the statistical program R version 4.1.0 and MicrobiomeAnalyst.^10^

## 3 RESULTS

The demographic and clinical characteristics of participants according to disease status are shown in Table 1. There was no significant difference in the family history of cardiovascular disease or the prevalence of diabetes and hypertension, or physical activity. As expected, body mass index (BMI) and blood triglycerides were higher in NAFLD patients (*p* < .0001 and *p* = .03, respectively). Women with PBC or NAFLD showed higher levels of transaminases and GGT than CTRL. PBC women had the highest ALP serum levels.

**TABLE 1:** Demographic and clinical variables of the 90 patients included in the study

Variable	PBC (30)	NAFLD (30)	CTRL (30)	*p*‐value	*p*‐value PBC vs. CTRL	*p*‐value NAFLD vs. CTRL	*p*‐value PBC vs. NAFLD
Age	59 (56–62.75)	55 (51–63.75)	55.5 (51.25–59)	.16	—	—	—
BMI (kg/m^2^)	22.85 (21.57–23.87)	28.4 (24.40–32.09)	22.50 (21.92–24.45)	**<.0001**	.8	**<.0001**	**<.0001**
Active smoking	4 (13.30)	10 (33.30)	5 (16.70)	.22	—	—	—
Hypertension	6 (20)	9 (30)	7 (23.30)	.75	—	—	—
Family history of CVD	16 (53.30)	18 (60)	15 (50)	.80	—	—	—
Sedentary	14 (46.70)	19 (63.30)	11 (36.70)	.14	—	—	—
Diabetes	—	5 (16.70)	—	**.009**	—	—	—
Liver stiffness (kPa)	5.40 (4.20–6)	4.50 (4–6.60)	—	—	—	—	—
UDCA (mg/day)	900 (787.5–900)	—	—	—	—	—	—
Total cholesterol (mg/dl)	225.5 (210.50–241.50)	234 (212.5–259.75)	230.5 (213.50–243.50)	.41	—	—	—
LDL (mg/dl)	144.5 (122.50–160.75)	157.5 (129–169.75)	138.5 (126.50–162)	.22	—	—	—
HDL (mg/dl)	64.5 (50.25–80)	55.5 (49–63.25)	62 (53.50–74.50)	.054	—	—	—
Triglycerides (mg/dl)	101 (76–122.5)	119.5 (92.25–147)	91 (78.5–112.75)	**.03**	**.04**	**.01**	.65
AST (IU/L)	27.5 (24–30)	22.5 (20–25)	17 (14.25–19.75)	**<.0001**	**<.0001**	**<.0001**	**.02**
ALT (IU/L)	26 (21–33)	24 (21–46.25)	15 (12–19.75)	**<.0001**	**<.0001**	**<.0001**	.99
ALP (IU/L)	128 (88.25–185.75)	86.5 (76–96.25)	66 (59.25–84.25)	**<.0001**	**<.0001**	**.04**	**.002**
GGT (IU/L)	42.5 (38–71)	29.5 (19.5–54)	14 (12–19)	**<.0001**	**<.0001**	**<.0001**	**.03**
Total bilirubin (mg/dl)	0.54 (0.4–0.77)	0.6 (0.5–0.77)	0.5 (0.37–0.67)	.09	—	—	—
PLTS (10^9^/L)	241.5 (209.75–265.25)	258 (221.75–327.75)	251 (229–285)	.25	—	—	—

Abbreviations: ALP, alkaline phosphatase; ALT, alanine transaminase; AST, aspartate transaminase; BMI, body mass index; CTRL, healthy controls; CVD, cardiovascular disease; GGT, gamma‐ glutamyl transferase; HDL, high‐ density lipoproteins; LDL, low‐ density lipoproteins; NAFLD, non‐alcoholic fatty liver disease; PBC, primary biliary cholangitis; PLTS, platelets.

*Note*: Upper limit of normal (ULN): AST = 45 IU/mL; ALT = 45 IU/mL; ALP = 116 IU/mL; GGT = 36 IU/mL. Liver stiffness was assessed by transient elastography.

Statistically significant comparisons are highlighted in bold.

### 3.1 Prevalence of atherosclerosis and expression of inflammatory and vasoactive mediators

**FIGURE 1:**
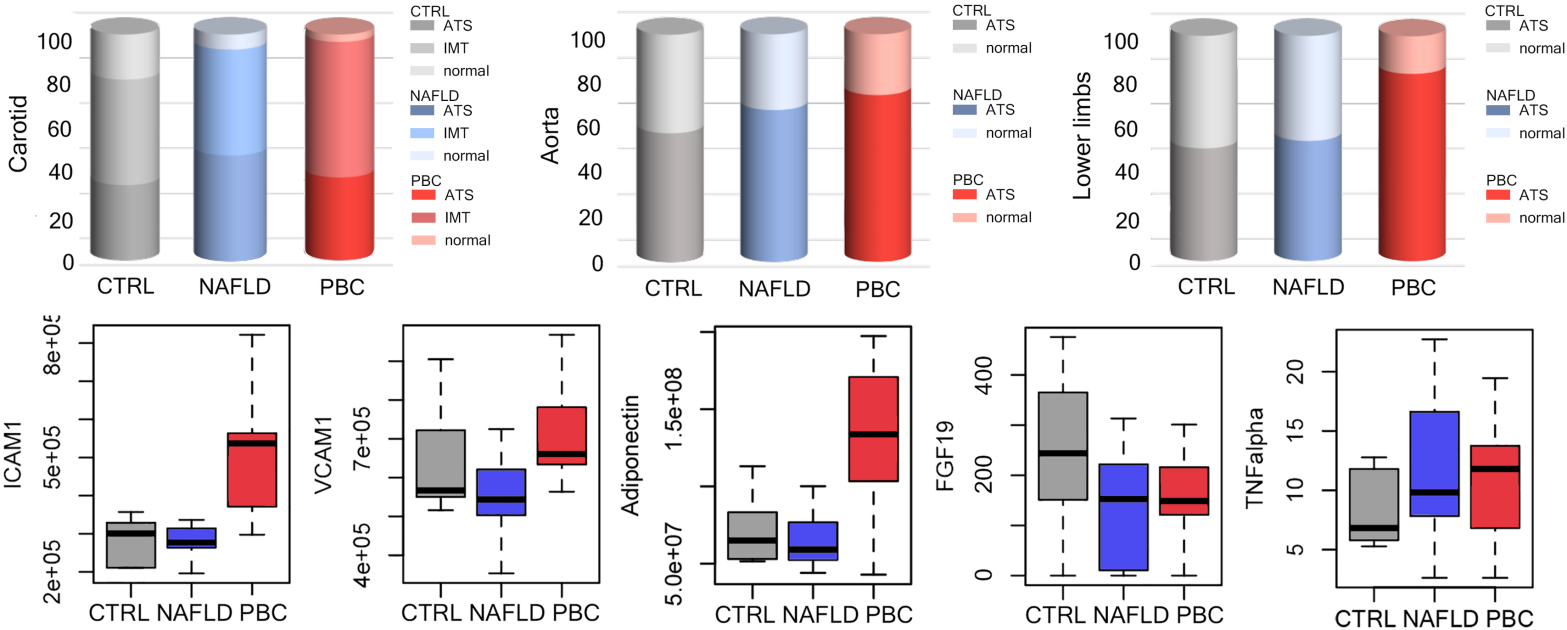
Prevalence of atherosclerosis in the different explored districts (carotids, abdominal aorta, lower limbs), FMD values, expression of cytokines, adhesion molecules, adiponectinand FGF19 between the study groups. CTRL, healthy controls; FGF19, fibroblast growth factor 19; ICAM‐1, intercellular adhesion molecule‐1; IFN, interferon; NAFLD, non‐alcoholic fatty liver disease; PBC, primary biliary cholangitis; TNF, tumourtumor necrosis factor; VCAM‐1, vascular adhesion molecule‐1.

At DUS examination, 46 (51.1%) participants had initial endothelial damage (intima‐media thickening) of the carotid arteries, whereas 35 (38.9%) had atherosclerotic plaques. Atherosclerosis of the aorta, including both parietal calcifications and fibrocalcific plaques, was found in 59 (65.6%) participants and LEAD in 56 (62.2%) participants. The prevalence of atherosclerotic lesions differed among groups, with LEAD being more frequent in PBC (83.34%) than in NAFLD (53.34%) or CTRL (50%) women (*p* = .01; Figure 1; Table 2). No differences in the prevalence of atherosclerosis of carotid arteries or aorta among groups were observed. Likewise, FMD was not significantly different among groups (Table 2).

**TABLE 2:** Prevalence of atherosclerosis between groups

Variable	PBC (30)	NAFLD (30)	CTRL (30)	*p*‐value	*p*‐value PBC vs. CTRL	*p*‐value NAFLD vs. CTRL	*p*‐value PBC vs. NAFLD
Carotid				.25	—	—	—
ATS	11 (36.70)	14 (46.70)	10 (33.30)				
IMT	18 (60)	14 (46.70)	14 (46.70)				
Normal	1 (3.30)	2 (6.60)	6 (20)				
Lower limbs				**.01**			
ATS	25 (83.34)	16 (53.34)	15 (50)				
Normal	5 (16.66)	14 (46.66)	15 (50)				
Aorta				.43	—	—	—
ATS	22 (73.33)	20 (66.66)	17 (56.66)				
Normal	8 (26.66)	10 (33.33)	13 (43.33)				
FMD (%)	11.70 (9.10–12.97)	9.90 (8.30–13.22)	10.30 (6.92–13.60)	.95	—	—	—

Abbreviations: ATS, atherosclerosis; CTRL, healthy controls; FMD, flow‐mediated dilation; IMT, intima media thickness; NAFLD, non‐alcoholic fatty liver disease; PBC, primary biliary cholangitis.

Statistically significant comparisons are highlighted in bold.

In the univariate analysis, factors associated with LEAD in the overall cohort were VCAM‐1 (*p* = .002), ICAM‐1 (*p* = .003) and TNF‐alpha (*p* = .04); in the multivariate regression model, VCAM‐1 (OR 1.1 95% CI 1.0–1.1; *p* = .04) and TNF‐alpha (OR 1.12, 95% CI 0.99–1.26; *p* = .04) were the only predictors of LEAD (Table 3).

**TABLE 3:** Univariate and multivariate analysis for factors predictive of lower‐extremity arterial disease (LEAD)

Factor	Univariate		Multivariate	
	OR (95% CI)	*p*‐value	OR (95% CI)	*p*‐value
BMI	1.04 (0.95–1.45)	.38	—	—
Diabetes	0.9 (1.07–2.57)	.92	—	—
VCAM‐1	1.000007 (1.000002–1.00001)	**.002**	1.000005 (1.0000004–1.00001)	**.03**
ICAM‐1	1.000007 (1.000002–1.00001)	**.003**	1.000004 (0.99–1.00001)	.18
FGF19	0.99 (0.99–1.000002)	.50	—	—
Adiponectin	1 (1–1)	.17	—	—
TNF‐alpha	1.11 (1.003–1.24)	**.04**	1.12 (1–1.25)	**.04**

Abbreviations: BMI, body mass index; CI, confidence interval; CTRL, healthy controls; FGF19, fibroblast growth factor‐19; ICAM‐1, intercellular adhesion molecule‐1; NAFLD, non‐alcoholic fatty liver disease; OR, odds ratio; PBC, primary biliary cholangitis; TNF, tumour necrosis factor; VCAM‐1, vascular adhesion molecule‐1.

Statistically significant comparisons are highlighted in bold.

Then, we explored whether a difference in the circulating levels of adhesion molecules and inflammatory cytokines was detectable in the three study groups. Serum levels of ICAM‐1, VCAM‐1 and adiponectin were significantly higher in PBC patients than in NAFLD patients and CTRL; FGF19 was lower in PBC and NAFLD women than in CTRL (Figure 1, Table S1). Notably, PBC women showed ICAM‐1, VCAM‐1, and adiponectin serum levels higher than NAFLD and CTRL women, even after removal of patients without LEAD. There was no difference among groups with regard to adipsin serum levels. As for inflammatory mediators, we observed higher serum levels of TNF‐alpha in PBC and NAFLD patients compared with CTRLs, but no differences in IL1‐beta, IL‐6 or IFN‐gamma.

### 3.2 Gut microbiota characterization

**FIGURE 2:**
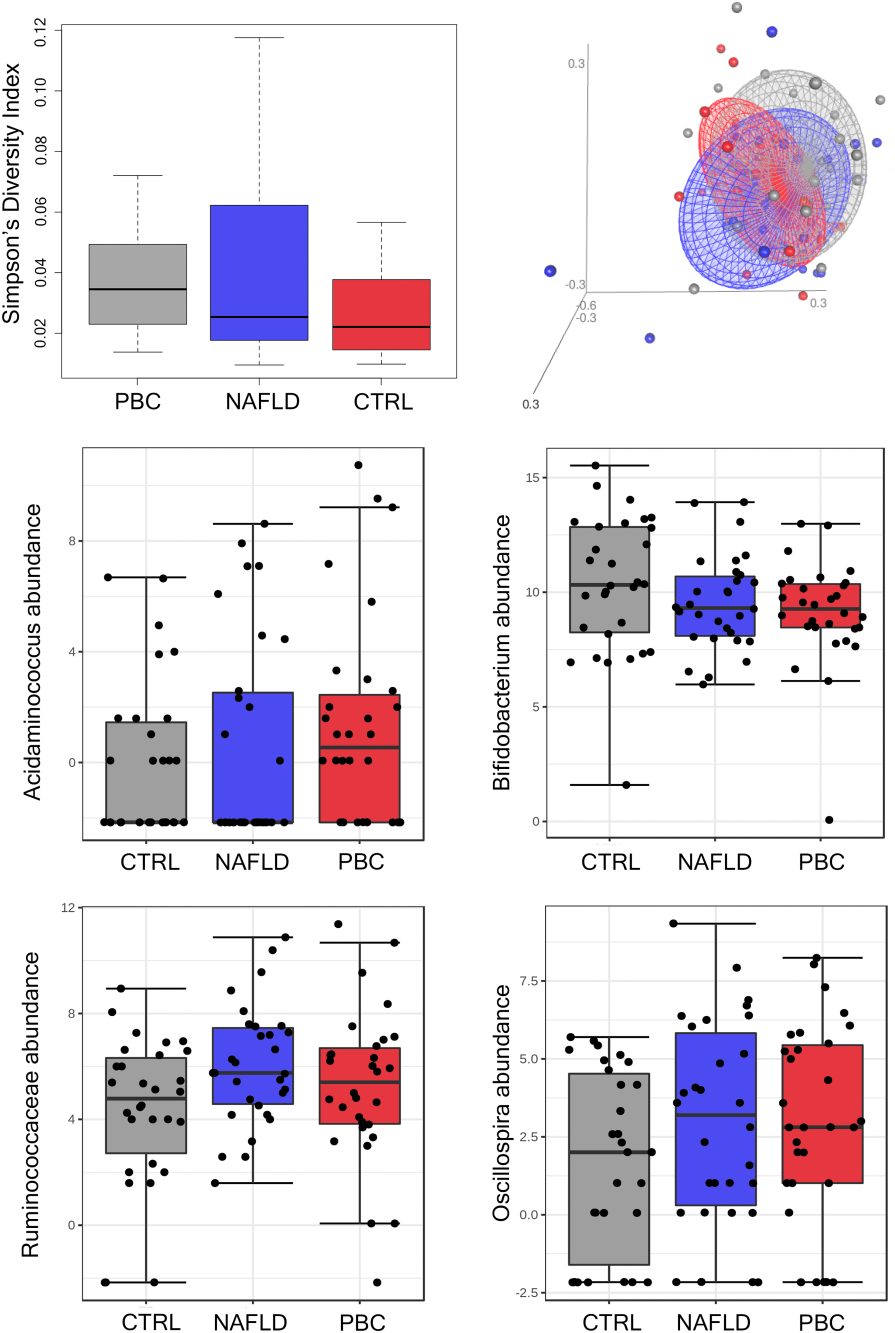
Gut microbiota analysis. Alpha diversity according to the Simpson's index and beta diversity (NMDS on Bray–Curtis distance) are shown in the upper left and right panel, respectively. Gut microbiota genera and families that mainly characterized PBC patients are also shown; bacterial abundance is reported as log‐transformed counts. CTRL, healthy controls; NAFLD, non‐alcoholic fatty liver disease; NMDS, non‐metric multidimensional scaling; PBC, primary biliary cholangitis.

We observed a slightly lower alpha‐diversity in PBC and NAFLD women than in CTRL (Simpson's index of 0.02 [0.015–0.04] vs. 0.025 [0.02–0.06] vs 0.03 [0.02–0.04], *p* = .14; Figure 2). As highlighted by NMDS based on Bray–Curtis distance, the three groups showed a different composition of the gut microbiota (PERMANOVA *p* = .03; Figure 2).

In particular, the relative abundance analysis revealed that *Acidaminococcus* (FDR = 0.0008), *Bifidobacterium* (FDR = 0.001) and *Oscillospira* (FDR = 0.03) were differentially expressed among groups (Table S2). Compared with controls, in PBC women *Acidaminococcus* (FDR = 0.005), *Ruminococcaceae* (FDR = 0.005) and *Oscillospira* (FDR = 0.008) were increased, whereas *Bifidobacterium* was reduced (FDR = 0.0006) (Figure 2). In NAFLD women, *Ruminococcaceae* (FDR = 0.002), *Oscillospira* (FDR = 0.01) and *Dialister* (FDR = 0.02) were increased, while *Clostridiales* (FDR = 0.02) and *Corynebacterium* (FDR = 0.04) were reduced. Finally, the difference between PBC and NAFLD consisted in the overabundance of *Streptococcus* (FDR <0.0001) and *Acidaminococcus* in the former group compared with the latter (FDR = 0.04).

**FIGURE 3:**
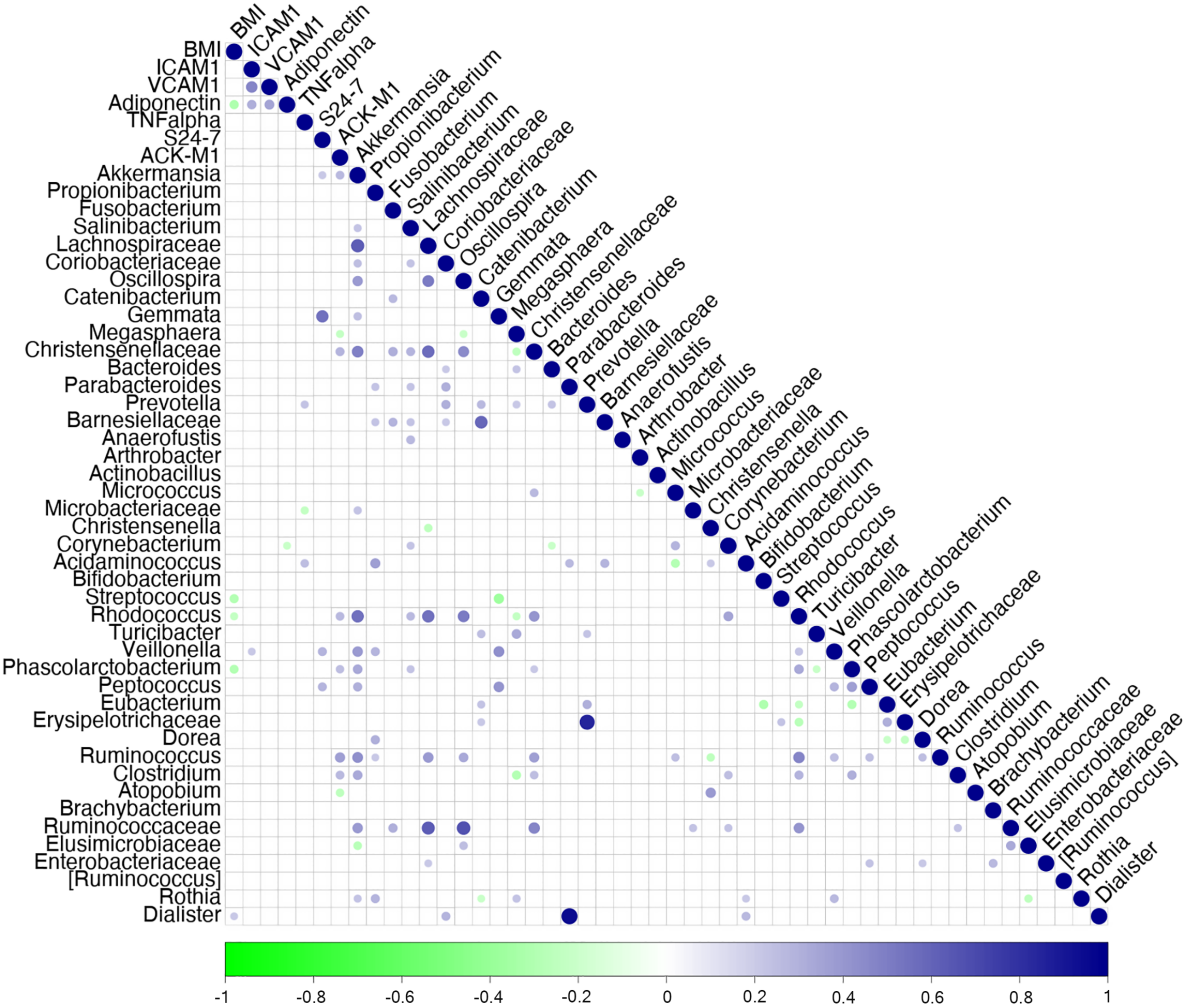
Correlations between cytokines, adhesion molecules and adipokines and the gut microbiota. Statistically significant correlations are represented by circles; positive ones are highlighted in blue and negative ones in green. The intensity of colour represents the strength of the correlation. BMI, body mass index; ICAM‐1, intercellular adhesion molecule‐1; IL, interleukin; TNF, tumour necrosis factor; VCAM‐1, vascular adhesion molecule‐1.

Regarding possible correlations between inflammatory and vascular mediators associated with atherosclerosis, *Acidaminococcus* (0.25, FDR = 0.02) and *Prevotella* (0.23, FDR = 0.03) had a positive correlation with TNF‐alpha, whereas *Microbacteriaceae* (−0.23, FDR = 0.03) had a negative correlation (Figure 3; Table S3). *Corynebacterium* had an inverse association with adiponectin (−0.21 FDR = 0.04) and *Veillonella* a positive correlation with ICAM‐1 (0.21 FDR = 0.04). No significant correlations were observed between VCAM‐1 and bacterial taxa.

### 3.3 Gut microbiota functional analysis

We finally analysed the predicted metabolic pathways of the functional gut metagenome to explore whether there was a possible implication with the increased prevalence of atherosclerosis in PBC women (Table 4). Compared with CTRL, several metabolic pathways were down‐regulated in PBC women, such as those involving lipid metabolism, that is, ‘fatty acid degradation’, ‘fatty acid metabolism’, ‘butanoate metabolism’, ‘synthesis and degradation of ketone bodies’, ‘glyoxylate and dicarboxylate metabolism’ and ‘valine, leucine and isoleucine degradation’.

**TABLE 4:** Gut microbiome metabolic pathways differentially expressed in the study groups. All pathways are down‐regulated compared with the reference group; only statistically significant results are reported

Pathway	Total	Expected	Hits	*p*val	FDR
PBC vs. CTRL					
Fatty acid degradation	39	1.15	9	<.0001	<0.0001
Butanoate metabolism	77	2.27	12	<.0001	<0.0001
Geraniol degradation	6	177	4	<.0001	<0.0001
Valine, leucine and isoleucine degradation	66	1.95	10	<.0001	<0.0001
Fatty acid metabolism	60	1.77	9	<.0001	0.00155
Caprolactam degradation	13	384	4	.000415	0.0109
Glyoxylate and dicarboxylate metabolism	68	2.01	8	.000734	0.0166
Synthesis and degradation of ketone bodies	8	236	3	.00125	0.0247
NAFLD vs. CTRL					
Butanoate metabolism	77	4.32	19	<.0001	<0.0001
Geraniol degradation	6	337	6	<.0001	<0.0001
Citrate cycle (TCA cycle)	57	3.2	16	<.0001	<0.0001
Valine, leucine and isoleucine degradation	66	3.71	17	<.0001	<0.0001
Carbon metabolism	299	16.8	39	<.0001	<0.0001
Pyruvate metabolism	86	4.83	18	<.0001	<0.0001
Fatty acid degradation	39	2.19	11	<.0001	0.000122
Propanoate metabolism	82	4.61	15	<.0001	0.000686
Phenylalanine metabolism	76	4.27	13	.00024	0.00421
Synthesis and degradation of ketone bodies	8	449	4	.000563	0.0089
Limonene and pinene degradation	5	281	3	.0016	0.023
Glycolysis/gluconeogenesis	82	4.61	12	.00176	0.0232
Styrene degradation	19	1.07	5	.00322	0.0391
Glyoxylate and dicarboxylate metabolism	68	3.82	10	.00408	0.0461
Caprolactam degradation	13	0.73	4	.00461	0.0485
PBC vs. NAFLD					
Biosynthesis of amino acids	223	8.8	31	<.0001	<0.0001
Peptidoglycan biosynthesis	17	671	8	<.0001	<0.0001
Pyrimidine metabolism	85	3.36	12	<.0001	0.00499
Streptomycin biosynthesis	15	592	5	.000193	0.00763
Purine metabolism	179	7.07	17	.00052	0.0164
D‐Glutamine and D‐glutamate metabolism	6	237	3	.0011	0.0275
Glycolysis/gluconeogenesis	82	3.24	10	.00122	0.0275

Abbreviations: CTRL, healthy controls; NAFLD, non‐alcoholic fatty liver disease; PBC, primary biliary cholangitis; TCA, tricarboxylic acid.

‘Pyruvate metabolism’, ‘citrate cycle (TCA cycle)’, ‘butanoate and propanoate metabolism’, ‘carbon metabolism and carbon fixation’, ‘cysteine and methionine metabolism’, ‘beta‐alanine metabolism’, ‘phenylalanine metabolism’ and ‘fatty acid degradation’ were down‐regulated in NAFLD group compared with CTRL. The main functional differences between PBC and NAFLD consisted in ‘biosynthesis of amino acids and peptidoglycan’, ‘purine and pyrimidine metabolism’, ‘D‐glutamine and D‐glutamate metabolism’ and ‘glycolysis/gluconeogenesis’.

## 4 DISCUSSION

Hypercholesterolemia is a common finding in PBC patients. Although previous studies did not show an increased risk of carotid atherosclerosis in PBC, our investigation revealed a high prevalence of LEAD, showing a correlation with the gut–liver axis.

To our knowledge, this is the first study that explored lower limb arteries in PBC patients, demonstrating that not all vascular districts are equally affected. Compared with the general female population of the same age, we found an almost doubled prevalence of LEAD in PBC women without associated clinical symptoms.^13^ This finding confirms the already reported risk of atherosclerosis in patients with autoimmune or inflammatory diseases.^3^, ^14^, ^15^ Based on these findings, PBC patients should deserve specific diagnostic investigations, that are not currently recommended by guidelines.

To further define the pathophysiology of LEAD in PBC, we studied the profile of circulating inflammatory and vasoactive mediators. Atherosclerosis is generally linked with inflammation and is characterized by increased systemic levels of cytokines and adhesion molecules.^16^ Indeed, we confirmed that serum levels of ICAM‐1, VCAM‐1, and TNF‐alpha were elevated in PBC women compared with those affected by NAFLD and CTRL. Previous studies reported an up‐regulation of ICAM‐1 and VCAM‐1 in portal tracts and sinusoidal endothelial cells of PBC patients, suggesting a role in lymphocyte recruitment.^17^ Nevertheless, the expression of ICAM‐1 and VCAM‐1 is enhanced by incubation with inflammatory cytokines, such as TNF‐alpha, as confirmed by our correlation analysis, and with lipopolysaccharide.^18^, ^19^ Therefore, the inflammatory response that is involved in the pathogenesis of biliary damage and cholestasis might also play a role in the development of vascular disease in PBC. We found higher adiponectin serum levels in PBC women compared with the other groups. As previously reported, adiponectin is increased in PBC and is considered a possible protective factor for the development of atherosclerosis.^20^ Its increase may reflect an attempt to counteract pro‐atherogenic mechanisms. Our group of PBC women also showed reduced levels of FGF19 than CTRL but similar to patients with NAFLD, probably due to optimal disease control and absence of cirrhosis; indeed, FGF19 serum levels are elevated in PBC non‐responders to UDCA or with advanced fibrosis, but decrease in UDCA responders.^21^ Among other activities, FXR is involved in the regulation of lipid metabolism, and its inactivation may promote atherogenesis.^22^ In the multivariate model, only VCAM‐1 and TNF‐alpha were independent predictors of LEAD. Thus, our results not only confirm previous data on the overexpression of adhesion molecules and enhanced inflammatory profile in PBC patients but also show a link with an extrahepatic complication, which may have a trigger similar to that of liver damage.

Imbalance of the gut–liver axis is a hallmark of liver disease and contributes to the progression of damage and the onset of complications. Modifications of the gut microbiota have been described in PBC patients, and previous studies also showed a connection between gut microbiota, hypercholesterolemia and atherosclerosis.^23^ Based on these premises and supported by the close association between TNF‐alpha and LEAD revealed by our analysis, we then characterized the gut microbiota of participants. We found an enrichment of *Acidaminococcus* in PBC women compared with the other groups, which had a direct correlation with TNF‐alpha serum levels. *Acidaminococcus* has been associated with a pro‐inflammatory diet and several disease markers.^24^, ^25^ Furthermore, its overabundance has already been reported in patients with carotid atherosclerosis and cardiovascular disease, and has been associated with TNF‐alpha production.^26^, ^27^, ^28^ The depletion of *Bifidobacterium*, which has known immunomodulatory properties,^29^ may contribute to the pro‐inflammatory condition of patients affected by PBC, although we were unable to demonstrate any correlation with the inflammatory or vasoactive mediators assayed. In addition, it should be considered that not only *Bifidobacterium* but also *Oscillospira*, which was enriched in PBC women, can deconjugate primary bile acids, altering the intestinal bile acids pool^30^, ^31^; therefore, their presence or absence can affect the activity of the FXR/FGF19 system. We also observed that *Ruminococcaceae* were increased in PBC women compared with CTRL, confirming previous findings.^32^

The gut microbiota is an important metabolic regulator, and this may be an additional mechanism by which its alteration may contribute to the development of LEAD in women affected by PBC. Compared with CTRL, we found a down‐regulation of metabolic pathways related to fatty acid degradation and metabolism. Fatty acid overload is associated with endothelial dysfunction through mechanisms involving oxidative stress and inflammation.^33^ In addition, butyrate production and its oxidation into ketone bodies were found to be reduced in PBC patients. As both butyrate and ketone bodies have shown to exert protective effects on the vascular system, this may further explain the increased prevalence of LEAD in PBC.^34^, ^35^ Other dysfunctional pathways involved glyoxylate and branched‐chain amino acids, which are linked with inflammatory and pro‐oxidative responses, cardiometabolic risk and atherosclerosis.^36^, ^37^

This study has limitations. First, only women with well‐controlled PBC were enrolled, so our findings should not be extended to men, conditions of advanced fibrosis/cirrhosis or to those non‐responding to UDCA. Nevertheless, ours is one of the largest Western cohorts of PBC patients in whom a gut microbiota analysis has been performed. Previous studies included Asian populations, small groups of patients without a control group or with other autoimmune disorders or advanced disease, making it difficult to compare results.^32^, ^38^, ^39^ In addition, our patients were all asymptomatic at the time of LEAD diagnosis, but no follow‐up data are available. Therefore, no inference can be made as to whether vascular alterations may be progressive and become clinically relevant over time. Another limitation is that metabolomic pathways were inferred from the gut metagenome, and the study lacks a metabolomic analysis on stool or blood samples.

In conclusion, women affected by PBC have a remarkably high prevalence of atherosclerosis in the lower limbs. This may be considered an extrahepatic manifestation of the disease possibly linked to the expression of vasoactive and inflammatory mediators, and to compositional and functional modifications of the gut microbiota. Based on our findings, it may be appropriate to plan a screening program of vascular complications in PBC patients and to implement preventive interventions targeting the gut–liver axis and systemic inflammation.

## FUNDING INFORMATION

The paper was supported by “Ministero della Salute ‐ Ricerca Corrente 2022”.

## CONFLICT OF INTEREST

None.

## Supporting information


Table S1

Table S2

Table S3

## Data Availability

The data that support the findings of this study are available on request from the corresponding author. The data are not publicly available due to privacy or ethical restrictions.
