# Supplementary material for: High prevalence of lower limb atherosclerosis is linked with the gut–liver axis in patients with primary biliary cholangitis
Source: Liver Int. 2022 Nov 11;43(2):370–80. doi: 10.1111/liv.15463 (PMC10100232; doi:10.1111/liv.15463)
Supplement: Supplementary file 1 — Table S1 [file LIV-43-370-s002.docx]

**Supplementary Table 2:** Expression of adhesion molecules and inflammatory mediators in the study population.

| **Variable** | **PBC**  **(30)** | **NAFLD**  **(30)** | **CTRL**  **(30)** | **p-value** | **p-value**  **PBC vs CTRL** | **p-value**  **NAFLD vs CTRL** | **p-value**  **PBC vs NAFLD** |
| --- | --- | --- | --- | --- | --- | --- | --- |
| **ICAM-1 (pg/mL)** | 536954.6  (371537.7-563408.9) | 276850.3  (264137.4-313362.2) | 301025.6  (212720.7-328900) | **<0.0001** | **<0.0001** | 0.48 | **<0.0001** |
| **VCAM-1 (pg/mL)** | 660897.4  (636794.5-780884.4) | 543732.9  (508306.5-620852.9) | 566753.2  (550016.6-690822.6) | **<0.0001** | **0.001** | **0.02** | **<0.0001** |
| **Adiponectin (pg/mL)** | 133580000  (104412500 -170710000) | 59051000  (52468000 -74970500) | 64919500  (53051000-83154000) | **<0.0001** | **0.0001** | 0.13 | **<0.0001** |
| **Adipsin (pg/mL)** | 1054300  (908920-1435450) | 953436  (611328-1099050) | 847514  (809075-1335100) | 0.24 | - | - | - |
| **FGF19 (pg/mL)** | 148.89  (122.69-214.01) | 152.81  (251.68-221.97) | 244.02  (153.97-358.52) | **0.001** | **0.004** | **0.0009** | 0.69 |
| **IL-1beta (pg/mL)** | 0.021  (0.0105000-0.021) | 0.021  (0.0017575-0.021) | 0.021  (0.0052575-  0.021) | 0.23 | - | - | - |
| **TNF-alpha (pg/mL)** | 11.8  (6.82-13.74) | 9.83  (7.83-16.37) | 6.82  (5.8-11.8) | **0.02** | **0.02** | **0.02** | 0.99 |
| **IL-6 (pg/mL)** | 0.47  (0.25-0.92) | 0.47  (0.01-0.92) | 0.01  (0.0025-1.15) | 0.23 | - | - | - |
| **IFN-gamma (pg/mL)** | 0.02  (0.02-0.09) | 0.05  (0.02-0.09) | 0.09  (0.00001-0.15) | 0.45 | - | - | - |

*PBC= primary biliary cholangitis; NAFLD= nonalcoholic fatty liver disease; CTRL= healthy controls; ICAM-1= intercellular adhesion molecule-1; VCAM-1= vascular adhesion molecule-1; FGF19= fibroblast growth factor 19; IL= interleukin; TNF= tumor necrosis factor; IFN= interferon*
